# Magnetic Nanoparticles with Dual Surface Functions—Efficient Carriers for Metalloporphyrin-Catalyzed Drug Metabolite Synthesis in Batch and Continuous-Flow Reactors

**DOI:** 10.3390/nano10122329

**Published:** 2020-11-24

**Authors:** Diána Balogh-Weiser, Balázs Decsi, Réka Krammer, Gergő Dargó, Ferenc Ender, János Mizsei, Róbert Berkecz, Benjámin Gyarmati, András Szilágyi, Róbert Tőtős, Csaba Paizs, László Poppe, György T. Balogh

**Affiliations:** 1 Department of Organic Chemistry and Technology, Budapest University of Technology and Economics, Műegyetem rkp. 3, H-1111 Budapest, Hungary; decsi.balazs@mail.bme.hu (B.D.); krareka@gmail.com (R.K.); poppe@mail.bme.hu (L.P.); 2 Department of Physical Chemistry and Materials Science, Budapest University of Technology and Economics, Műegyetem rkp. 3, H-1111 Budapest, Hungary; bgyarmati@mail.bme.hu (B.G.); aszilagyi@mail.bme.hu (A.S.); 3 Department of Chemical and Environmental Process Engineering, Budapest University of Technology and Economics, Műegyetem rkp. 3, H-1111 Budapest, Hungary; gergo.dargo@gmail.com; 4 Department of Electron Devices, Budapest University of Technology and Economics, Műegyetem rkp. 3, H-111 Budapest, Hungary; ender.ferenc@vik.bme.hu (F.E.); mizsei.janos@vik.bme.hu (J.M.); 5 SpinSplit Llc., Vend u. 17, H-1025 Budapest, Hungary; 6 Institute of Pharmaceutical Analysis, Faculty of Pharmacy, University of Szeged, Somogyi utca 4., H-6720 Szeged, Hungary; berkecz.robert@pharm.u-szeged.hu; 7 Biocatalysis and Biotransformation Research Center, Faculty of Chemistry and Chemical Engineering, Babeş-Bolyai University of Cluj-Napoca, Arany János str. 11, 400028 Cluj-Napoca, Romania; totos.robert@yahoo.com (R.T.); csaba.paizs@ubbcluj.ro (C.P.); 8 Institute of Pharmacodynamics and Biopharmacy, Faculty of Pharmacy, University of Szeged, Eötvös u. 6, H-6720 Szeged, Hungary

**Keywords:** magnetic nanoparticles, metalloporphyrin, biomimetic oxidation, drug metabolism, microfluidic chip reactor

## Abstract

The dual functionalization of magnetic nanoparticles with inert (methyl) and reactive (aminopropyl) groups enables efficient immobilization of synthetic metalloporphyrins (such as 5,10,15,20-tetrakis(2,3,4,5,6-pentafluorophenyl)iron(II) porphyrin and 5,10,15,20-tetrakis-(4-sulfonatophenyl)iron(II) porphyrin) via covalent or ionic interactions. The proportion of reactive function on the surface has significant effect on the biomimetic activity of metalloporphyrins. The optimized magnetic nanocatalyst containing porphyrin was successfully applied for biomimetic oxidation of antihypertensive drug Amlodipine in batch and continuous-flow reactors as well.

## 1. Introduction

Nanotechnology is one of the fastest developing fields of science. The importance of this area is obvious, with its advances not only being used in biotechnology [1], the food industry [2], pharmacology [3] and the construction industry [4]. One promising use of nanotechnology is the development and application of nanomaterials. According to the classic definition, nanomaterials are a type of materials, that have, at least in one dimension, or have a structural component in size range 1–100 nm. Among nanomaterials, nanoparticles have a prominent role in many fields, due to their greater surface to mass ratio compared to bulk materials [5]. The big specific surface area is a benefit that makes nanoparticles ideal for carriers of reactants and catalysts in heterogeneous reactions or active drugs for specific treatments [6].

Magnetic nanoparticles (MNPs) are a specific group of nanomaterials. While they exhibit all the beneficial properties of nanoparticles, such as large specific surface area and they are ease of functionalization with organic or inorganic molecules. In addition, MNPs show paramagnetic behavior thus, there is a unique opportunity for control and separation of them by magnetic field. Thus, MNPs can be an ideal carrier material in many applications, especially in catalytic reactions [7]. Different particle sizes and narrow size distribution can be achieved by fine tuning the synthesis conditions. Thereby MNPs offer a unique solution for targeted therapeutic treatments (magnetic drug targeting, hyperthermia of tumor cells), and feasible contrast agent in MRI diagnostics [8].

With a view applying MNPs as carrier material, their surface usually should normally be coated and/or modified to prevent any possible toxic leakage of ions during their biological use. Moreover, the bare particles do not present reactive groups to immobilize compounds on their surface and tend to aggregate when dispersed in solvents. To achieve surface modification, there are two possible methods. The precursor of the coating agent can be introduced in-situ during particle formation or after the synthesis of MNPs. Different organic (chitosan, dextran, polyethylene glycol, etc.) or inorganic (gold, silica, etc.) materials can be used for coating the surfaces of MNPs [9].

Coating of MNPs with silica layer offers easily modifiable surface silanol groups, which can be used to establish reactive groups on the surface of MNPs. One of the most common solutions is the modification with a silica layer which can be obtained readily on MNP cores by Stöber method, commonly performed with tetraethoxysilane (TetraEthyl OrthoSilicate: TEOS). TEOS is hydrolyzed in aqueous alcoholic medium using ammonium hydroxide as a catalyst to provide a continuous nanoporous silica network [10]. Coating MNPs with a silica layer produces easily modifiable surface silanol groups, thereby enabling subsequent modifications that will create further reactive groups on the MNPs. Most commonly amino groups–introduced by surface-coating with amino group containing organosilanes, like 3-aminopropyltrimethoxy or triethoxysilane [11] are used as reactive sites at the surface. Amino functions can be further modified in order to create new types of reactive functional groups like carboxy [12] or hydroxyl groups [13].

Because of their magnetic properties, as well as their good chemical and mechanical stability, MNPs are very attractive catalyst-carriers in synthetic reactions. For example, palladium-based Suzuki-coupling agents were immobilized on polymer- and silica-coated nanoparticles or rhodium-based selective catalyst was anchored with polyaminoamido dendrons on silica-coated MNPs and was used for hydroformylation reactions. The supported catalyst had high reactivity and selectivity [14]. MNPs are viable carriers for biocatalysts like enzymes as well. In our previous work, a recombinant phenylalanine ammonia lyase from *Petrosenillium crispum* (*Pc*PAL) was immobilized on MNPs and was used to catalyze deamination of acyclic amino acids [15] or selectively immobilized on MNPs with metal ion affinity and covalent functions [16].

The study of the metabolism of drug candidates is one of the most important issues to consider when it comes to discovering new drugs. The main routes of drug metabolism are based on enzymatic biotransformations which are often started by an oxidative step, catalyzed by the cytochrome P450 (CYP, CYP450) isoenzyme family [17]. To characterize the possible metabolic fate of a drug candidate in the early preclinical development, in vitro metabolic stability studies are carried out. Usually, in these tests, hepatocyte and/or liver microsome-based methods are used due to their pharmacokinetic relevance and high concentrations of CYP enzymes therein. However, in these cell/microsome-based experiments biological matrix is formed with too big complexity, due to the necessity of coenzymes and their regeneration systems. The complexity of the matrix causes difficult metabolite analysis and allows only quantitative analysis of the compounds; moreover, the high cost and weak stability of hepatocyte and microsome-based methods pose remarkable limitations [18]. To improve these results, liver microsomes were immobilized on magnetic nanoparticles with amine-functions via secondary forces and used them in synthesis of diclofenac metabolites. But the low applied substrate concentration and serious microsome desorption limit the usability of the microsome-MNP system [19,20].

In order to tackle the drawbacks of in vitro hepatocyte- or microsome-based methods, in vitro biomimetic process were introduced as a promising alternative. For these kinds of biomimetic systems, the main goal is to be able to synthetize metabolites from parent molecule in a single step.

With a view to realising such a system, synthetic metalloporphyrins are used as biomimetic catalysts. The applicability of metalloporphyrins is based on their structural similarity with the prosthetic heme group within active site of the CYP enzymes. Synthetic metalloporphyrins proved to be feasible catalysts to generate different metabolites under various reaction conditions [21,22,23,24]. The drawbacks of using metalloporphyrins are their fast degradation in homogeneous oxidative systems and the difficulties of isolating them from the products. To improve the stability and recovery of porphyrin catalysts in such systems, metalloporphyrins can be immobilized on solid carriers. The immobilization can be achieved by covalent bond or by secondary interactions such as ionic bond or hydrogen bond. In our previous study, we anchored meso-tetra(pentafluorophenyl)iron porphyrin by covalent bond and meso-tetra(4-sulphonatophenyl)iron porphyrin by ionic bond onto simple aminopropyl-grafted MNPs. These immobilized catalyst systems were used under continuous-flow reaction conditions to synthesize Amiodarone metabolites [25].

**Figure 1:**
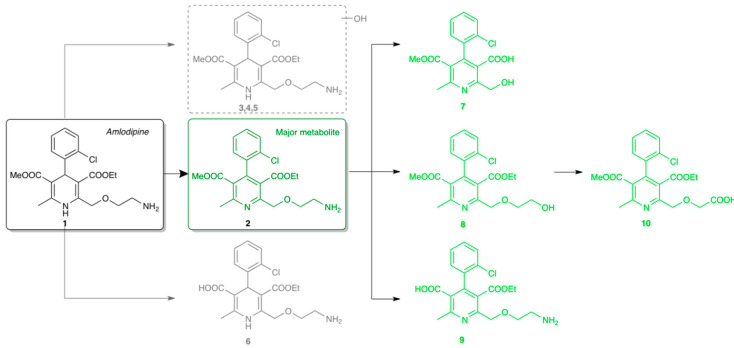
Amlodipine and its metabolites in human liver microsome.

Amlodipine (**1**, Figure 1) is a dihydropyridine derivative calcium channel blocker, which inhibits the so-called slow channel influx of calcium ion into cardiac and vascular tissue and has a vasodilatory effect in the peripheral vasculature and in the coronary vascular beds [26]. Amlodipine was introduced by Pfizer to the pharmaceutical market in 1990 under the brand name Norvasc and since 2007 it has been available as a generic drug. In clinical studies, amlodipine shows a quite long elimination of half-life (~35 h) after a single 10 mg intravenous dose and it is metabolized slowly but extensively in the liver. The first metabolic step of amlodipine is the oxidation of the dihydropyridine moiety to the pyridine analogue (Figure 1). 

The major metabolite (**2**, Figure 1) and its derivatives that stem from oxidative deamination, *O*-demethylation and *O*-dealkylation are major drug-related components (**7**–**10**, Figure 1) in human urine. The previously described metabolite profile suggest that amlodipine dehydrogenation to **2** followed by multiple oxidative transformations of **2** is the major hepatic clearance pathway of Amlodipine in humans and metabolic routes are very similar in rats and dogs as well. In vitro and in vivo studies also showed that the major metabolite of amlodipine is the dehydrogenated pyridine analogue (**2**, Figure 1) which is primarily related to the CYP3A4 activity and none of the pyridine derivative metabolites of amlodipine have significant pharmacological activity [27]. However this metabolic route is not common, for example CYP-related metabolism of nonsteroidal antimineralocorticoid finerenone shows a similar oxidative pathway [28].

**Figure 2:**
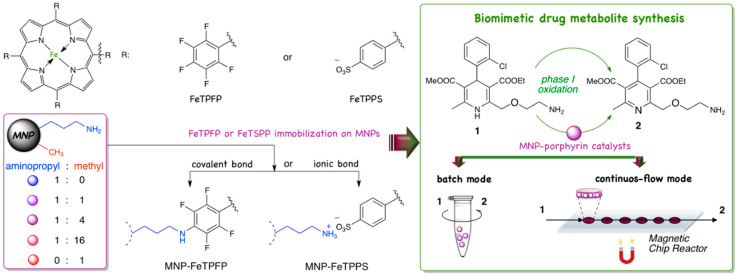
Covalent and ionic immobilization of porphyrins (FeTPFP and FeTPPS) on aminopropyl/methyl grafted magnetic nanoparticles (MNPs) and the application of the MNP-porphyrin in biomimetic oxidation of amlodipine **1** (forming didehydro-amlopdipine metabolite **2**) in batch mode and in continuous-flow Magnetic Chip Reactor (MCR).

Our previous studies showed that the nature of the functional groups, the density of reactive groups and the hydrophobic/hydrophilic properties of catalyst carriers can be well-controlled by surface modifications by means binary and ternary mixtures of substituted organosilanes. The systematic combination of organosilanes provided improved immobilized biocatalysts built up from nanoparticles or nanoporous networks [16,28,29,30,31]. MNPs with dual functions created by surface modification with binary mixtures of organosilanes (containing aminopropyl-trimethoxy silane:methyl-trimethoxysilane mixtures at molar ratios of 1:0, 1:1, 1:4, 1:16 and 0:1) had not previously been investigated yet for the immobilization of metalloporphyrins. In this paper we optimized the surface properties of MNPs for covalent and ionic binding of 5,10,15,20-tetrakis(2,3,4,5,6-pentafluorophenyl)iron(II) porphyrin (FeTPFP) and 5,10,15,20-tetrakis-(4-sulfonatophenyl)iron(II) porphyrin (FeTPPS) catalysts. The MNP-porphyrin catalysts were applied in the biomimetic oxidation of Amlodipine in batch and continuous-flow mode for the first time (Figure 2).

## 2. Materials and Methods

### 2.1. Materials

All solvents used in this experiments were of analytical grade. Methanol (MeOH), 2-propanol, formic acid, acetonitrile (AcN), acetic acid, sodium hydroxide, *n*-propanol and diglyme were the product of Merck Ltd. (Budapest, Hungary). Water was obtained from a Millipore (Bedford, MA, USA) Milli-Q water-purification system and applied for the preparation of all aqueous solutions. Amlodipine, *t*-butyl hydroperoxide (*t*BuOOH), iron (III) chloride × 6 H_2_O (FeCl_3_), sodium-acetate × 3 H_2_O, polyethylene glycol 400 (PEG 400), polyethylene glycol 4000 (PEG 4000), ethylene glycol, tetraethoxysilane (TEOS), 3-aminopropyltrimethoxysilane, methyl-trimethoxysilane and Ninhydrin were purchased from Sigma-Aldrich (St. Louis, MO, USA). Metalloporphyrins, such as 5,10,15,20-tetrakis(2,3,4,5,6-pentafluorophenyl)iron(II) porphyrin (FeTPFP) and 5,10,15,20-tetrakis-(4-sulfonatophenyl)iron(II) porphyrin (FeTPPS), were purchased from Frontier Scientific (Logan, UT, USA).

### 2.2. Methods

#### 2.2.1. Synthesis of Magnetic Nanoparticles (MNPs)

Iron(III) chloride-hexahydrate (20.2 g) was sonicated in ethylene-glycol (600 mL) for 10 min. Polyethylene glycol (PEG) 4000 (20.2 g) and sodium acetate trihydrate (54.0 g) were added to the mixture, then it was sonicated until it produced a homogeneous solution (~10 min). The mixture was intensively stirred in a stainless-steel autoclave for 24 h at 200 °C. After magnetic separation (applying permanent neodymium magnets, N35) the MNPs were washed three-times with water (~300 mL, each) and two-times with ethanol (~300 mL, each) and were dried in a vacuum cabinet (at room temperature, 1.5 mbar) until constant mass.

#### 2.2.2. Coating of MNPs with Silica Layer (MNPs-TEOS)

MNPs (5.0 g) and PEG 400 (5.0 g) were added to the mixture of ethanol (125 mL) and water (25 mL) and the suspension was sonicated for 20 min. Ammonia solution (12.5 mL, 35% aqueous solution) was added to the suspension and was shaken for 5 min at room temperature. Tetraetoxysilane (TEOS, 7.5 mL) was added to the suspension and the resulted mixture was shaken for 24 h at room temperature. After magnetic separation (applying permanent Neodymium magnets, N35), the MNP-TEOS was washed three-times with water (~20 mL, each) and two-times with ethanol (~20 mL, each) and was dried in vacuum cabinet (at room temperature, 1.5 mbar) until constant mass.

#### 2.2.3. Surface Functionalization of MNPs-TEOS with Binary Mixture of Aminopropyltrimethoxy-silane and Methyltrimethoxysilane (Ap/Me-MNPs)

MNPs-TEOS (250 mg) were added to the mixture of PEG 400 (50 mg) and ethanol (5.0 mL) and the suspension was sonicated for 30 min. Ammonia solution (25 µL, 35%) was added to the suspension and the mixture was sonicated for 10 min at room temperature. Mixture of the binary silane precursors (1 mmol; with different molar ratio of 3-aminopropyl-trimethoxysilane and methyl-trimethoxysilane n:n = 1:0, 1:1, 1:4, 1:16, 0:1) in ethanol (5.0 mL) was added to the suspension and was shaken for 24 h at room temperature. After magnetic separation (applying permanent Neodymium magnets, N35), the functionalized MNPs were washed three-times with distilled water (~5 mL, each) and two-times with ethanol (~5 mL, each) and were dried in vacuum cabinet (at room temperature, 1.5 mbar) until constant mass.

#### 2.2.4. Covalent Immobilization of FeTPFP on Functionalized MNPs (MNPs-FeTPFP)

Functionalized magnetic nanopartciles (Ap/Me-MNPs, 10.0 mg) was added to diglyme (7.0 mL) and was sonicated for 30 min. Solution of 5,10,15,20-tetrakis(2,3,4,5,6-pentafluorophenyl)iron(II) porphyrin (FeTPFP) in diglyme (1 mL, 0.5 mg mL^−1^) was added to the suspension which was shaken for 72 h at 60 °C. After magnetic separation (applying permanent Neodymium magnets, N35), the FeTPFP-MNPs were washed with isopropanol (~5 mL), distilled water (~5 mL), and methanol (~5 mL) and were dried in vacuum cabinet (at room temperature, 1.5 mbar) until constant mass.

#### 2.2.5. Ionic Immobilization of FeTPPS on Functionalized MNPs (FeTPPS-MNPs)

Functionalized magnetic nanoparticles (Ap/Me-MNPs, 10.0 mg) were added to methanol:sodium acetate buffer mixture (4:1 v/v, 7.0 mL, pH = 4.5) and sonicated for 10 min. Then a solution of 5,10,15,20-tetrakis-(4-sulfonatophenyl)iron(II) porphyrin (FeTPPS) in methanol:acetate buffer mixture (4:1 v/v, 8.0 mL, pH = 4.5 (1.0 mL, 0.5 mg/mL) was added to the suspension which was shaken for 15 min at room temperature. After magnetic separation (applying permanent Neodymium magnets, N35), the FeTPPS-MNPs were washed three-times with methanol (~5 mL, each), then dried in vacuum cabinet cabinet (at room temperature, 1.5 mbar) until constant mass.

#### 2.2.6. Ninhydrin Assay for Determination of Amin-Content of Functionalized MNPs

Functionalized magnetic nanoparticles (3.0 mg) were added to sodium acetate buffer (300 µL, 100 mM, pH = 5.5) which were sonicated for 5 min to get dispersed suspension. Ninhydrin reagent solution (600 µL, 3% w/v in n-propanol) was added to the suspension and was shaken for 15 min at 100 °C. After magnetic isolation, the upper phase (0.9 mL) was analyzed by a Genesys 2 type ultraviolet-visible (UV-VIS) spectrophotometer (Thermo Fisher Scientific Inc., Waltham, MA, USA) at 30 °C. The specific absorbance was determined at 570 nm wavelength. The calibration curve with 3-aminopropyltrimethoxysilane was also recorded.

#### 2.2.7. Zeta-Potential Analysis

The zeta potential of naked, silica coated, amino- and inert group-functionalized MNPs was measured with a Zeta Potential Analyzer (Brookhaven, Holtsville, NY, USA) using the Zeta Phase Analysis Light Scattering (PALS) method. Re-dispersed samples (0.4 mL, containing 1.0 mg nanoparticles and 1.0 mL 1 mM KCl) were diluted 5-fold in 1 mM KCl aqueous solution. Measurements carried out in a disposable, solvent resistant micro cuvette and took 2 min. The zeta potential was calculated from the electrophoretic mobility using the Smoluchowski equation.

#### 2.2.8. Analysis of Particle Size Distribution

Particle size distribution of naked, silica coated, amino- and inert group-functionalized MNPs were characterized by dynamic light scattering (DLS, Brookhaven BI-200SM Laser Light Scattering Instrument, Holtsville, NY, USA). The particles (ca. 2 mg) were sonicated in ethanol (6.0 mL) for 20 min, than analyzed by a laser beam (λ = 488 nm) at 25 °C in three parallel runs.

#### 2.2.9. Immobilization Yield of MNP-Porphyrins

After the immobilization process of metalloporphyrins (FeTPFP or FeTPPS), a sample (900 µL) taken directly from the residual binding solvent freed from Ap/Me-MNPs was analyzed by a Genesys 2 type ultraviolet-visible (UV-VIS) spectrophotometer (Thermo Fisher Scientific Inc., Waltham, MA, USA) at room temperature. The specific absorption wavelength (λ_max_) of the corresponding metalloporphyrin was determined (λ_max_ = 407 nm for FeTPFP and λ_max_ = 395 nm for FeTPPS), then calibration curves of metalloporphyrins in methanol:sodium acetate buffer mixture (4:1 v/v, 7.0 mL, pH = 4.5) were also recorded. The immobilization yield (Y_I_, %) was calculated from the following equation:

(1) $$Y_{I}=(1-\frac{c_{2P}}{c_{1P}})\times \;100$$

where $c_{1P}$ is the initial metalloporphyrin concentration, $c_{2P}$ is the residual metalloporphyrin concentration in the binding solution.

#### 2.2.10. General Method of Homogeneous Biomimetic Oxidation of Amlodipine Catalyzed by Non-Immobilized Metalloporphyrin in Batch Mode

Amlodipine solution (675 µL, 1.24 mg mL^−1^ in methanol), acetate buffer (200 µL, acetate buffer pH = 4.5), metalloporphyrin solution (125 µL, 1.23 mg mL^−1^ in methanol for FeTPFP and FeTPPS as well or 125 µL methanol without porphyrin as a blank reaction) and oxidizing agent solution (*t*BuOOH in water, 70%, 2 µL) was pipetted in an Eppendorf tube (with size 1.5 mL) and the resulting mixture was shaken for 30 min at 37 °C. The reaction mixture (0.5 mL) was analyzed by HPLC-DAD or HPLC-DAD-MS method described in Section 2.2.12 and Section 2.2.13.

#### 2.2.11. General Method of Biomimetic Oxidation of Amlodipine Catalyzed by Immobilized Metalloporphyrin on Functionalized Magnetic Nanoparticles in Batch Mode

In Eppendorf tubes (with size 1.5 mL) a mixture of porphyrin-carrying magnetic nanoparticles (2.0 mg), amlodipine solution (675 µL, 1.24 mg/mL in methanol), acetate buffer (200 µL, acetate buffer pH = 4.5) and methanol (125 µL) was sonicated for 20 min. The reaction was started by the addition of the oxidizing agent solution (*t*BuOOH in water, 70%, 2 µL). The reaction mixtures were shaken at 37 °C (for 1 h in the case of MNPs-FeTPFP and for 15 min with MNPs-FeTPPPS). After magnetic separation (applying permanent magnets of MagnaRack^TM^ magnetic separation rack from Invitrogen^TM^, ThermoFisher Scientific, Waltham, MA, USA see in Figure S1), the clear phase (0.5 mL) was analyzed by HPLC-DAD or HPLC-DAD-MS method described in Section 2.2.12 and Section 2.2.13 (see in Supplementary Materials Sections 1.1 and 1.2).

#### 2.2.12. General Method of the Microfluidic Biomimetic Oxidation

The loading of the reactor and the biomimetic oxidation of amlodipine was carried out by Magneflow system (SpinSplit LLC, Budapest, Hungary) as follows. The suspension of MNPs-FeTPPS (FeTPPS immobilized on amino:methyl 1:4 grafted MNP, 5.0 mg mL^−1^ in methanol) was driven through (flow rate: v = 0.01 mL min^−1^) the chip and the particles were anchored in the six reaction chambers by permanent magnets (neodymium spot magnets, N35) according to our previous studies (see also Figure 4). [22] The MNPs-FeTPPS catalyst content in any individual reaction chamber (regular cylindrical chamber with diameter 3600 µm and height 500 µm) was cc. 200 µg, thus the chip reactor contained 1.2 mg catalyst in its six reaction chambers. The filling process was observed by a high-speed USB camera (ARTCAM-500MI CMOS, Artray, Tokyo, Japan) see in Figure S2A) and the saturation of chambers was investigated by a digital microscope (BX51M, Olympus, Hong Kong, China, equipped with a MPlanFL-N 5×objective, see in Figure S2B). Amlodipine (cAmlodipine = 0.5, 0.25 or 0.125 mg mL^−1^ in methanol:sodium acetate buffer, 4:1 v/v, pH = 4.5) and oxidizing agent (tBuOOH, 5 equiv., in methanol:sodium acetate buffer, 4:1 v/v, pH = 4.5, 64 mM) solution were driven through the MNP loaded chip reactor at different flow rates (v = 0.1, 0.2 and 0.3 mL min^−1^) following a pre-programmed sequence of SpinStudio software (SpinSplit LLC, Budapest, Hungary). A timeframe of 15 min was provided to ensure the reach of equilibrium before samples were taken. The samples were analyzed by HPLC-DAD or HPLC-DAD-MS technique described in Section 2.2.12 and Section 2.2.13. The structure of the produced metabolite (2) was investigated by HRMS (see in Section 2.1 and Section 2.2, Table S1, Figure S4).

#### 2.2.13. Liquid Chromatography (LC) Method for Determination of Amlodipine and Its Metabolite

For a rapid investigation of porphyrin catalyzed biomimetic oxidation of amlodipine was performed on an Agilent 1100 liquid chromatography system equipped a diode array detector (DAD) (Agilent Technologies, Palo Alto, CA, USA). Chromatographic analysis was performed on a Kinetex^®^ 2.6 µm C18 100 Å column (30 × 3.0 mm) at 45 °C. Composition of mobile phases, eluent A and B (flow rate 1.0 mL min^−1^): eluent A was 0.1% (v/v) formic acid in water, eluent B was AcN/water 95/5 (v/v) with 0.1% (v/v) of formic acid. A 2.7 min long, linear gradient program was applied: 20% B in the first 0.2 min, 20–60% B between 0.2–1.2 min, then 60% B was kept for another 0.5 min, and finally at 1.71 min the percentage of B was dropped to 20%. This was followed by an equilibration period of 1.0 min prior to the next injection. Chromatograms were recorded at 220 ± 4 nm (injection volume was 1 µL). The retention times of amlodipine and its metabolite were 1.77 min and 1.61 min, respectively. ChemStation A.10.02 was used for data acquisition and analysis.

#### 2.2.14. Liquid Chromatography Coupled to Mass Spectrometry (LC-DAD-MS) Parameters for Determination of Amlodipine and Its Metabolite

Experiments were carried out on an Agilent 1200 liquid chromatography system coupled with an 6410 QQQ-MS (Agilent Technologies), equipped with DAD. Analysis was performed at 45 °C on a Kinetex EVO C18 column (50 × 3 mm, 2.6 µm, Phenomenex, Torrance, CA, USA). Composition of mobile phases eluent A and B (flow rate of 1.45 mL min^−1^): eluent A was 0.1% (v/v) trifluoroacetic acid (TFA) in water (pH 1.9), eluent B was a mixture of acetonitrile and water in 95:5 (v/v) with 0.1% (v/v) TFA. A linear gradient of 2–100% B was applied at a range of 0–4.9 min, then 100% B at 4.9–6.0 min. It was followed by a 1.20 min equilibration period prior to the next injection. The injection volume was set at 5 µL and the chromatographic profile was registered at 220 ± 4 nm. The mass spectrometer detector (MSD) operating parameters were as follows: electrospray ionization (ESI) positive ionization, scan ion mode (*m/z* 100–900), drying gas temperature 350 °C, nitrogen flow rate 11 L min^−1^, nebulizer pressure 40 psi, quadrupole temperature 100 °C, capillary voltage 4000 V, fragmentor voltage 135 V. A representative LC-MS chromatogram and MS spectra of biomimetic oxidations can be seen in Figures S4–S6).

#### 2.2.15. Liquid Chromatography Coupled to High-Resolution Mass Spectrometry (LC-HRMS/MS) Parameters for Determination of Amlodipine and Its Metabolite

An LC-HRMS/MS analysis was performed using a Waters Acquity I-Class UPLC™ (Waters, Manchester, UK). The UHPLC system was coupled to a Thermo Scientific Q Exactive Plus hybrid quadrupole–Orbitrap (Thermo Fisher Scientific) mass spectrometer. The LC-HRMS/MS method for the analysis of Amlodipine and its dehydrogenated metabolite was the following: Acquity UPLC HSS C18 column (100 mm × 2.1 mm × 1.8 µm, Waters), injection volume 5 µL and column temperature 50 °C. Mobile phase A was 0.1% formic acid in water and 0.1% formic acid in acetonitrile. The following eluent and flow rate gradient program was used: 0 min, 10% B (0.4 mL min^−1^); 4 min, 60% B (0.4 mL min^−1^); 5 min, 100% B (0.6 mL min^−1^); 6 min, 100% B (0.6 mL min^−1^); 6.1 min, 10% B (0.6 mL min^−1^); 9.7 min, 10% B (0.6 mL min^−1^); 9.71 min, 10% B (0.4 mL·min^−1^); and 10 min, 10% B (0.4 mL min^−1^). The mass spectrometer was operated in full scan and parallel reaction monitoring acquisition (PRM) modes using a heated ESI source with the following parameters: capillary temperature 262.5 °C, S-Lens RF level 50, spray voltage 3.5 kV, sheath gas flow 50, spare gas flow 2.5 and auxiliary gas flow 12.5. Mass range was set at 300–600 *m/z* (full scan) with a resolution of 70,000 (full scan) and 17,500 (PRM). The automatic gain control (AGC) setting was defined as 3 × 106 (full scan) and 1 × 106 (PRM) charges and the maximum injection time was set to 100 ms (full scan) and 30 ms (PRM). Collision energy and isolation window were set to 20 eV (amlodipine), 25 eV (dehydro amlodipine) and 2 *m/z* in PRM mode. The HRMS spectra of biomimetic oxidations can be seen in Table S1 and Figure S7.

#### 2.2.16. Nuclear Magnetic Resonance (NMR) Measurements

NMR data were acquired on a 500 MHz Avance III HD spectrometer (Bruker, Billerica, MA, USA) equipped with a Prodigy BBO or TCI cryogenically cooled probe head, respectively. Chemical shifts are reported in ppm referenced to TMS (^1^H) or residual solvent signals (3.31/49.15 ppm for ^1^H/^13^C in case of CD_3_OD or 2.505/39.5 ppm for ^1^H/^13^C in case of DMSO-d_6_). Standard one- and two-dimensional NMR data were acquired all cases at 298 K using standard pulse sequences available in the Topsin 3.5 sequence library. Data analysis and reporting were accomplished by ACD/ NMR Workbook 2015.2.9.

#### 2.2.17. Calculation of Biomimetic Reaction Parameters

The conversion of the substrate (*c*, %), biomimetic activity (*U*_B_, U/g), specific activity (*U*_P_, U g^−1^), and space time yield (STY, g L^−1^ h^−1^) were calculated by using the following equations based on LC-DAD chromatograms recorded at 220 ± 4 nm:

(2) $$c\;\left[\%\right]=(1-\frac{n_{S}}{n_{S}+n_{P}})\;\times 100$$

where *n*_S_ and *n*_P_ are the molar amounts of substrate (*S*) and product(s) (*P*),

(3) $$U_{B}\left[{U\;g}^{-1}\right]=\frac{\left(n_{S0}\times c\right)}{\left(t\times m_{B}\right)}$$

where *U* is rate of the substrate conversion [µmol min^−1^], *n*_S0_ is the initial amount of the substrate, *c* is conversion, *t* is reaction time in min, *m*_B_ is the mass of the biomimetic catalyst in g,

(4) $$U_{P}\left[{U\;g}^{-1}\right]=\frac{\left(n_{S0}\times c\right)}{\left(t\times m_{P}\right)}$$

where *m*_P_ is the mass of the metalloporphyrin in g in the immobilized biomimetic catalyst

(5) $$STY\left[{g\;L}^{-1}{\;h}^{-1}\right]=\frac{m_{p}}{V_{r}\times t}$$

where *m_p_* is the mass of the products in g, *V_r_* is the volume of the reactor in L and *t* is time in h.

## 3. Results

### 3.1. Characterization of Magnetic Nanoparticle Carriers with Different Aminopropyl- and Methyl- Functionalized Surface

The magnetic nanoparticles covered by a silica layer were functionalized with binary mixtures of aminopropyl- and methyltrimethoxysilane. In binary mixture the molar ratios of aminopropyl and methyl silane were chosen as following: 1:0 (pure aminopropyl covered surface), 1:1, 1:4, 1:6 and 0:1 (pure methyl covered surface). The amino-group content, the electrokinetic potential (zeta-potential) and immobilization efficiency (by the determination of immobilization yield, *Y*_I_) for FeTPFP- and FeTPPS-porphyrins (via covalent and ionic interactions respectively) on the various dual functionalized MNPs were investigated (Table 1). As expected from the number of amino-function group of the different MNPs, the increasing concentration of the methylsilane in the binary mixture decreased the density of the aminopropyl function at the surface. These results are consistent with the immobilization yield values for covalent immobilization of FeTPFP. The electrokinetic potential, which can be related to colloidal stability of the MNP suspensions, also showed similar trend, by the decreasing concentration of amino-groups the positive zeta-potential shifted to negative. The immobilization yields of the two types of metalloporphyrins indicated that the efficiency of FeTPPS immobilization was much better than that of FeTPFP. This difference can be justified by the different way of immobilization (covalent or ionic binding), while the activation energy requirement is higher in case of the covalent binding. In addition, the different solubilities of the two metalloporphyrins can be also affected on the immobilization process.

### 3.2. Investigation of Metalloporphyrins (FeTPFP and FeTPPS) for Biomimetic Oxidation of Amlodipine

As a preliminary study, 5,10,15,20-tetrakis(2,3,4,5,6-pentafluorophenyl)iron(II) porphyrin (FeTPFP) and 5,10,15,20-tetrakis-(4-sulfonatophenyl)iron(II) porphyrin (FeTPPS) were investigated in biomimetic oxidation of Amlodipine (1) and reactions without porphyrin (solubilized or immobilized on magnetic nanoparticles) with *t*BuOOH oxidizing agent (see in Section 2.2.10). HPLC-DAD measurements showed that with FeTPFP catalyst and FeTPPS catalyst conversions 29.5 ± 1.5 and 99.5 ± 0.4% were achieved respectively. In addition, in case of blank samples, which did not contain porphyrin catalysts no Amlodipine transformation were observed.

### 3.3. Biomimetic Oxidation of Amlodipine Catalyzed by FeTPFP Metalloporphyrin Immobilized on Dual-Grafted MNPs (FeTPFP-MNPs) in Batch Mode

**Figure 3:**
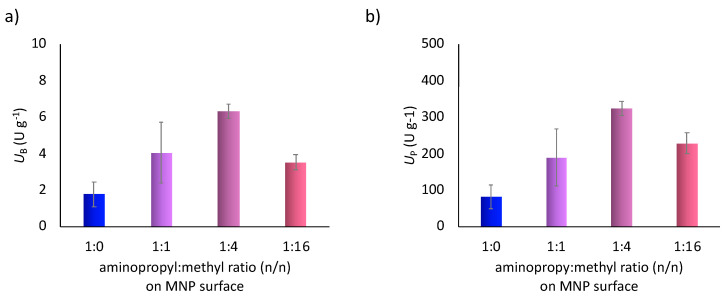
Effect of the aminopropyl:methyl ratio at the surface of MNPs on (**a**) the biomimetic activity (U_B_) and on (**b**) the specific activity (U_P_) b) of the MNPs-FeTPFP catalyts in biomimetic oxidation of Amlodipine (**1**).

The covalent immobilization of FeTPFP porphyrin was performed by using four MNP carriers with differently grafted surfaces (the molar ratios of aminopropyl:methyl groups were 1:0, 1:1, 1:4 and 1:16). The catalytic activities of the various FeTPFP-MNPs were compared in the biomimetic oxidation of amlodipine (1) applying *t*BuOOH as oxidizing agent in simple shaken vial reactions in batch mode (Figure 3. Analysis of the reactions by LC-MS (according to Section 1.1 and Section 1.2 in Supplementary Materials) could only detect the human major metabolite, dehydro-amlodipine (2). The biomimetic activity (*U*_B,_ which means the amount of the converted substrate per minutes catalyzed by one gram of the immobilized catalyst) and the specific activity (*U*_P,_ which means the amount of the converted substrate per minutes catalyzed by one gram of the porphyrin catalyst) data for the FeTPFP porphyrin immobilized at various dual-functionalized MNPs indicated that the MNP carriers grafted with aminopropyl:methyl functions at 1:4 molar ratio provided the best results in both cases. It can also be recognized that the effect of surface density of the aminopropyl group showed the highest levels, as a trend. While the immobilization yields were not significantly divergent (see in Table 1), the significantly different catalytic activities could be attributed to the altered FeTPFP-densities (resulting in various porphyrin to porphyrin distances) as a dominant parameter.

### 3.4. Biomimetic Oxidation of Amlodipine Catalyzed by FeTPPS Metalloporphyrin Immobilized on Dual Grafted MNPs (FeTPPS-MNPs) in Batch Mode

**Figure 4:**
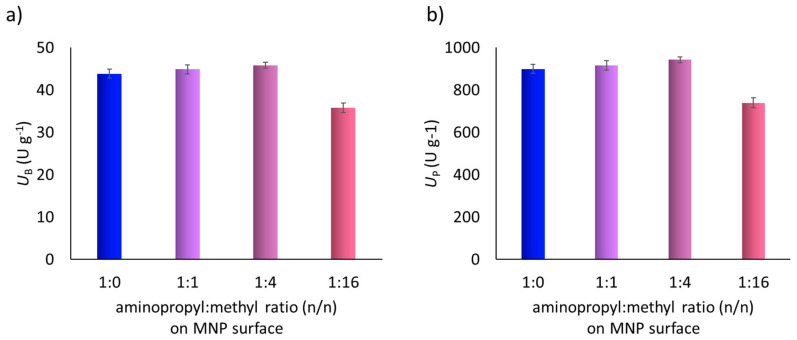
Effect of the aminopropyl:methyl ratio of MNPs surface on (**a**) the biomimetic activity (U_B_) and on the (**b**) specific activity (U_P_) of MNP-FeTPPS catalyts in biomimetic oxidation of amlodipine (**1**).

In case of ionic immobilization of FeTPPS porphyrin, the same MNPs with grafted surfaces were applied as carriers (MNPs functionalized by aminopropyl-:methyltrimethoxysilane mixtures at 1:0, 1:1, 1:4, 1:16 and 0:1 n:n). According to the results in Table 1., four MNPs-FeTPPS catalysts were tested similarly as described in the case of FeTPFP-MNPs in biomimetic oxidation of amlodipine (1) (Figure 4). The reaction led only to formation of the major human metabolite (2) according to the LC-MS analysis, similarly to the FeTPFP-MNP-catalyzed reactions. It was shown that the aminopropyl:methyl ratio had no significant effect on the immobilization yield of the FeTPPS porphyrin (Table 1), as in the case of FeTPFP. Notable difference in the immobilization efficiency (Table 1) among the investigated MNPs carriers was not found. Apparently, all investigated MNP-carriers were able to anchor the total amount of FeTPPS porphyrin from the binding solution. The influence of the surface properties on catalytic activity was much less pronounced than in the case of the various FeTPFP-MNPs. Only a very slight trend with an activity maximum at the aminopropyl:methyl 1:4 grafting ratio could be observed, but there were no serious differences between the 1:0, 1:1 and 1:4 precursor mixtures used for MNPs modification. This observation can be explaineded by taking into account the fewer binding possibilities of FeTPPS (by means of the sulfonic acid moieties) than of the FeTPFP (by nucleophilc replacement of the numerous fluorine substituents). Thus, the effects of surface polarity and of amino-group density cannot prevail enough to influence the catalytic properties of the metalloporphyrin. However, it is notable, the biomimetic activity (*U*_B_) of the FeTPPS-MNPs catalysts was more than five-times bigger than that of the corresponding MNPs-FeTPFP.

### 3.5. Continuous-Flow Biomimetic Oxidation Catalyzed by FeTPPS-MNPs in a Microfluidic Magnetic Chip-Reactor

**Figure 5:**
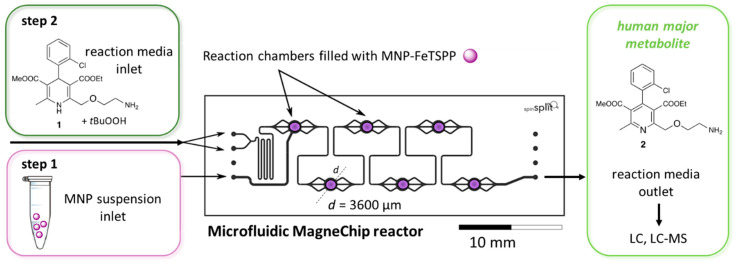
Schematic layout of biomimetic oxidation of amlodipine (**1**) catalyzed by MNPs-FeTPPS (FeTPPS porphyrin immobilized on aminopropyl:methyl 1:4 grafted MNPs by ionic binding) in continuous-flow microfluidic MagneChip reactor.

For the continuous-flow experiments a microfluidic magnetic chip reactor (MagneChip, SpinSplit Llc., Budapest, Hungary) was applied. First, the six consecutive reaction chambers were filled with the suspension of FeTPPS-MNPs (FeTPPS immobilized on aminopropyl:methyl 1:4 grafted MNPs by ionic binding) as catalyst (Figure 5, step 1). Then the reaction media containing amlodipine (**1**) as model drug and *t*-BuOOH as oxidizing agent were fed into the chip reactor (Figure 5, step 2) by a syringe pump.

The concentration of amlodipine (**1**) (c_Amlodpine_ = 0.5, 0.25 and 0.125 mg mL^−1^) and the flow rate of the reaction media (v = 0.1, 0.2 and 0.3 mL min^−1^) were systematically changed for optimization of the biomimetic oxidation. The effluent reaction media from the chip reactor after steady state were analyzed by LC and LC-MS similarly to the batch reactions. Results showed that the MagneChip reactor exhibited a behaviour typical of traditional continuous-flow reactors (e.g., simple packed bed reactors), while with increased flow rate or substrate (by meaning Amlodipine) concentration the conversion value decreased.

**Figure 6:**
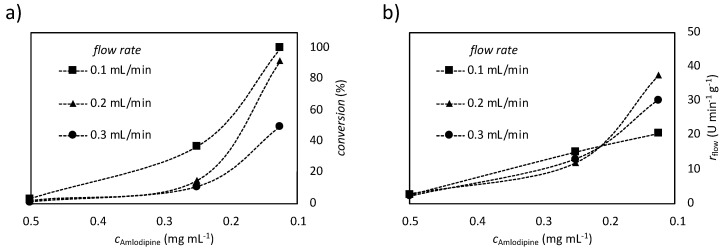
Biomimetic oxidation of amlodipine catalyzed by FeTSPP immobilized on MNPs (MNPs are grafted with aminopropyl:methyl 1:4) in a continuous-flow magnetic-chip reactor, effect of the flow rate and Amlodipine concentration on the (**a**) conversion and (**b**) productivity (r_flow_).

Total conversion of amlodipine (**1**) into the major human liver metabolite, dehydro-amlodipine (**2**) could be reached at 0.1 mL min^−1^ flow rate and 0.125 mg mL^−1^ substrate concentration (Figure 6a). The productivity of the reactor (r_flow_) was optimal at the same substrate concentration (c_Amlodipine_ = 0.125 mg mL^−1^) but higher flow rate (v = 0.2 mL min^-1^) (Figure 6b).

### 3.6. Comparison of the Space Time Yield (STY) Values of the Biomimetic Reactions

For a reliable comparison of the different process based on the different forms and applicaton modes of metalloporphyrin catalysts (homogenous batch, heterogenous batch and heterogenous continuous-flow), Space Time Yield values were calculated (Table 2). STY is a generally applied and informative parameter for the comaparison of catalytic systems applied in different processes (batch or continuous) [32]. STY data clearly showed that the immobilized forms (FeTPFP-MNPS and FeTPPS-MNPs) were comparable to the non-immobilized homogenous form of metalloporphyrin catalysts. Moreover, efficiency of the immobilized FeTPPS-MNPs in the continuous-flow magnetic chip reactor was significantly higher than that of the corresponding batch reaction performed in shaken vial.

## 4. Discussion

This study showed immobilization of metalloporphyrin on magnetic nanoparticles (MNPs) as a promising way to perform effective biomimetic catalysis. To demonstrate the applicability of the MNP-based forms, two commonly used iron porphyrins, 5,10,15,20-tetrakis(2,3,4,5,6-pentafluoro-phenyl)iron(II) porphyrin (FeTPFP) and 5,10,15,20-tetrakis-(4-sulfonatophenyl)iron(II) porphyrin (FeTPPS) were selected as catalyst for the biomimetic oxidation of amlodipine as a popular antihypertensive drug molecule to produce the major human metabolite. The structure of metalloporphyrins influenced the catalytic effectivity in their various forms. While FeTPFP-catalyzed reactions reached higher STY in homogenous form than as immobilized on MNPs, FeTPPS was more active as immobilized catalyst on MNPs. This was even more apparent when the specific biomimetic activities of the two immobilized forms were compared: FeTPPS-MNPs had more than ten times higher *U*_B_ (67.2 U g^−^^1^) than the *U*_B_ for FeTPFP-MNPs (6.3 U g^−^^1^). 

A major finding of this report is the serious effect of surface properties of MNPs–such as the density of functional groups and the type of interactions between the carrier and metalloporphyrin (covalent or ionic)–on the final biomimetic capability on the immobilized metalloporphyrin catalysts. It was shown that inert functions (like methyl-groups) as surface modifiers can control the density and localization of reactive functional groups (such as primary amine) which can directly interact with a proper part of a metalloporphyrin molecule. By fine-tuning the binary surface etching mixture which contains inert (methyltrimethoxysilane) and reactive (3-aminopropyltrimethoxysilane) silane components, the surface properties can be optimized for immobilization of different metalloporphyrins resulting in magnetic nanocatalysts with enhanced activity. The optimized novel magnetic nanocatalyst (FeTPPS-MNPs) was successfully applied in a microfluidic magnetic chip reactor operated in continuous-flow mode with more than ten times higher Space Time Yield (25.5 g l^−^^1^ h^−^^1^) than what was achieved in a shake vial system in batch (2.25 g l^−^^1^ h^−^^1^) for biomimetic production of the human metabolite didehydro-amlodipine (**2**) of amlodipine (**1**) with total conversion after 15 min residence time. The quality of produced metabolite (**2**) was verified by HRMS (see in Supplementary Material Section 2.2, Table S1 and Figure S4), comparing to literature [33] and NMR measurements (see in Supplementary Material Section 2.2) The two orders of magnitude higher STY values of magnetic catalyst integrated in a magne-chip reactor as compared to the shake vial system demonstrated the outstanding usefulness of the chip-sized magnetic flow-reactor system. 

## 5. Conclusions

Fast and robust production of drug metabolites is a very important issue in the early stages of drug discovery but also in clinical candidate selection and drug development. The main disadvantages of traditional in vivo or cell-based metabolite synthesis can be avoided by using biomimetic systems based on special organic frameworks with red-ox catalytic activity like metalloporphyrins. The evolution of metalloporphyrin catalysts can be promoted by well-designed immobilization technique and smart carriers. Magnetic nanoparticles are suitable carriers to immobilize metalloporphyrins giving access to easy-to-use catalysts and clean reaction media after straightforward catalyst-separation. Fine-tuning of surface properties of MNPs can be easily achieved by dual-function etching with reactive and inert functions, which provide access to novel catalysts with significantly enhanced catalytic activity. Use of MNP-bound metalloporphyrins in chip-sized continuous-flow reactors applied for effective biomimetic metabolite synthesis can strongly contribute to the pre-clinical drug investigations.

## Figures and Tables

**Table 1:** Comparison of the amino-function content, the electrokinetic potential (zeta-potential) and immobilization efficiency (*Y*_I_) of the MNPs carriers grafted with different aminopropyl:methyl ratio at the surface.

Aminopropyl:Methyl Molar Ratio on MNP Surface (n/n)		Mean Particle Diameter (nm)	Quantity of Amino Function ^a^ (µmol mg^−1^)	Zeta-Potential (mV)	*Y*_I_^b^ (%)	
					for FeTPFP	for FeTPPS
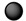	bare MNP^c^	186	<0.2	16.7 **±** 5.6	n.d. ^d^	n.d. ^d^
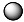	MNP coated by TEOS ^e^	218	<0.2	−40.9 ± 0.9	n.d. ^d^	n.d. ^d^
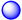	1:0	231	3.6 ± 0.4	18.5 ± 1.5	42.7 ± 0.9	97.4 ± 0.4
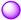	1:1	329	3.5 ± 0.3	10.4 ± 0.8	42.7 ± 0.7	98.0 ± 0.6
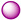	1:4	370	1.4 ± 0.2	−1.5 ± 0.1	39.3 ± 0.5	97.3 ± 0.6
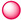	1:16	335	0.9 ± 0.1	−6.8 ± 0.5	30.7 ± 0.7	96.9 ± 0.4
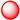	0:1	225	<0.2	−35.6 ± 2.7	n.d. ^d^	n.d. ^d^

^a^ quantity of the amino groups (µmol) on one unit of MNP carrier (mg) measured by ninhydrin-assay, ^b^ *Y*_I_: immobilization yield (proportion of the porphyrin immobilized on MNP carrier), ^c^ naked MNP (without silica coating or further modification), ^d^ not detectable quantity, ^e^ non-functionalized MNP (coated by silica shell without further modification).

**Table 2:** Comparison of STY values of biomimetic systems in the oxidation of amlopdipine catalyzed by metalloporphyrins.

Mode of Biomimetic Oxidation	Homogenous Batch		Heterogenous Batch		Continuous-Flow
	FeTPFP	FeTPPS	FeTPFP-MNPs	FeTPPS-MNPs	FeTPPS-MNPs
STY (g L^−1^ h^−1^)	0.49	1.67	0.31	2.25	25.5

## Supplementary Materials

The following are available online at https://www.mdpi.com/2079-4991/10/12/2329/s1, Figure S1: Illustration of magnetic separation of MNP-catalysts (surface grafted MNPs covered by metalloporphyrin FeTPFP or FeTPPS). The MNP-catalysts were used in biomimetic oxidation of Amlodipine (1) in batch mode. FigureS2: Photo of MNP-catalysts filled chip of continuous-flow Magnetic Chip reactor prepared by high speed USB camera A) and digital microscopic image of MNP-catalyst filled chamber B). Figure S3: Determination of particle size distribution of magnetic particles at different stages of modification by DLS: MNP (naked magnetic particles), MNP-TEOS (MNP coated by silica layer by TEOS), MNP-TEOS-Am (MNP-TEOS surface grafted by pure amino-functions), MNP-TEOS-Am/Me 1/1 (MNP-TEOS surface grafted by amino- and methyl-functions at molar ratio 1/1), MNP-TEOS-Am/Me 1/4 (MNP-TEOS surface grafted by amino- and methyl-functions at molar ratio 1/4), MNP-TEOS-Am/Me 1/16 (MNP-TEOS surface grafted by amino- and methyl-functions at molar ratio 1/16), MNP-TEOS-Me (MNP-TEOS surface grafted by pure methyl-functions). Figure S4: Representative HPLC data of FeTPFP-MNPs-catalyzed biomimetic oxidation of amlodipine (1) to major in vivo didehydro metabolite (2) in batch mode. Figure S5: MS spectrum of amlodipine (1). Figure S6: MS spectrum of amlodipine didehydro metabolite (2) as a main product of biomimetic oxidation. Figure S7: HRMS\MS spectrum of amlodipine didehydro metabolite (2) as a main product of biomimetic oxidation. Table S1: Summary of retention time, observed parent- and characteristic fragment ions of amlodipine and didehydro amlodipine.
