# Supplementary material for: Magnetic Nanoparticles with Dual Surface Functions—Efficient Carriers for Metalloporphyrin-Catalyzed Drug Metabolite Synthesis in Batch and Continuous-Flow Reactors
Source: Nanomaterials (Basel). 2020 Nov 24;10(12):2329. doi: 10.3390/nano10122329 (PMC7759782; doi:10.3390/nano10122329)
Supplement: Supplementary file 1 [file nanomaterials-10-02329-s001.pdf]

Supplementary Materials: Magnetic Nanoparticles with Dual Surface Functions—Efficient Carriers for Metalloporphyrin-Catalyzed Drug Metabolite Synthesis in Batch and Continuous-Flow Reactors

Diána Balogh-Weiser [1,2,*], Balázs Decsi [1], Réka Krammer [1], Gergő Dargó [3], Ferenc Ender [4,5], János Mizsei [4], Róbert Berkecz [6], Benjámin Gyarmati [2], András Szilágyi [2], Róbert Tőtős [7], Csaba Paizs [7], László Poppe [1,7] and György T. Balogh [3,8,*]

1. Characterization of Magnetic Carriers and Catalysts

1.1. Magnetic Separability of MNP-Catalysts in Batch Mode

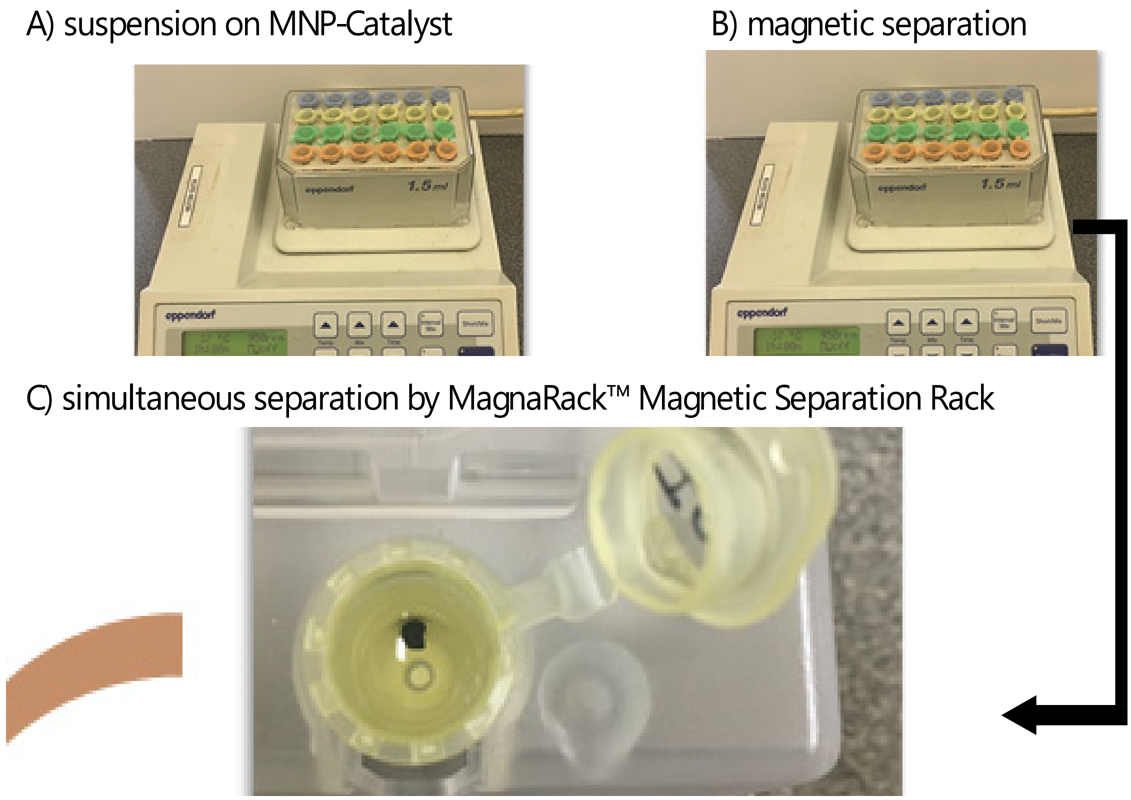

Figure S1. Illustration of magnetic separation of MNP-catalysts (surface grafted MNPs covered by metalloporphyrin FeTPFP or FeTPPS), stable suspension of MNP-catalyst **A**), magnetic separation of MNP-catalyst by permanent magnet **B**) and the parallel separation of MNP-catalysts by MagnaRack containing permanent magnets **C**). The MNP-catalysts were used in biomimetic oxidation of Amlodipine (**1**) in batch mode.

1.2. Magnetic Separability of MNP-Catalysts in Continuous-Flow Mode

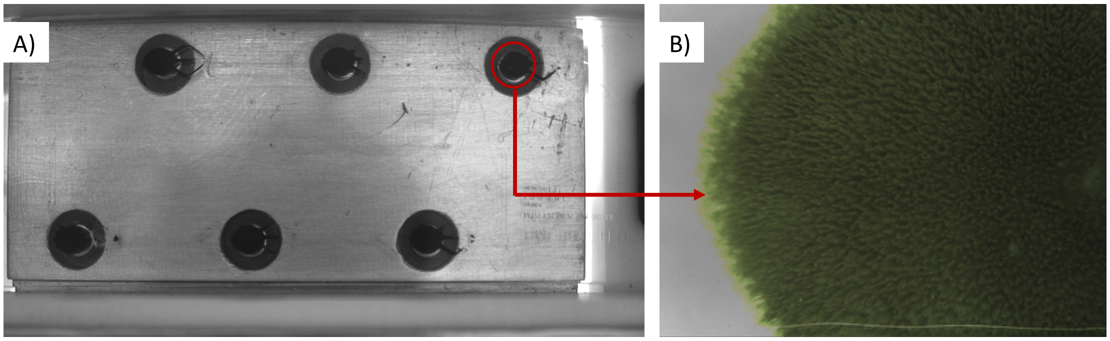

Figure S2. Photo of MNP-catalysts filled chip of continuous-flow Magnetic Chip reactor prepared by high speed USB camera **A**) and digital microscopic image of MNP-catalyst filled chamber **B**).

1.3. Results of DLS Measurement of Magnetic Particles at Different Stages of Modifications

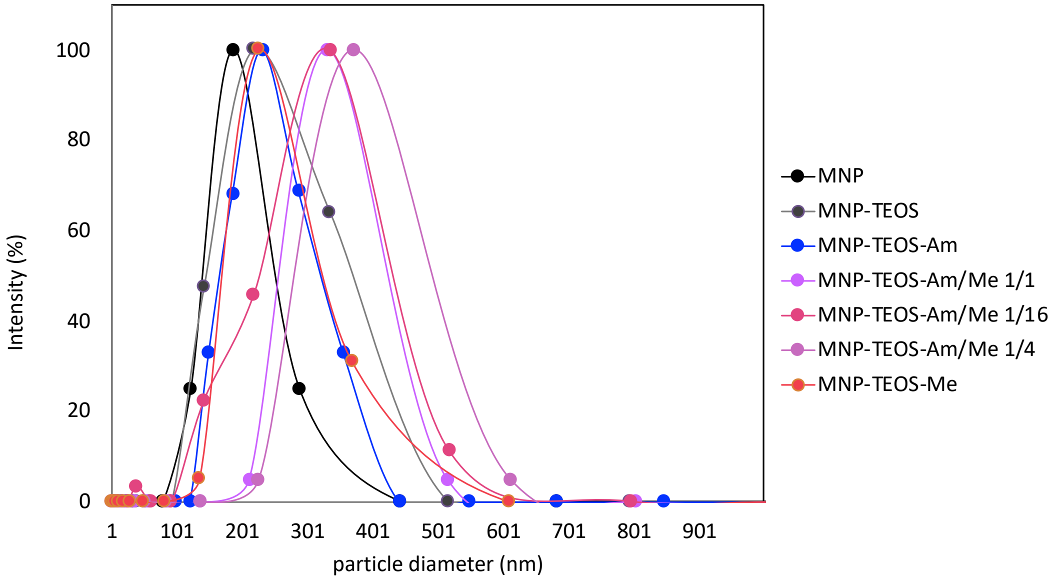

Figure S3. Determination of particle size distribution of magnetic particles at different stages of modification by DLS: MNP (naked magnetic particles), MNP-TEOS (MNP coated by silica layer by TEOS), MNP-TEOS-Am (MNP-TEOS surface grafted by pure amino-functions), MNP-TEOS-Am/Me 1/1 (MNP-TEOS surface grafted by amino- and methyl-functions at molar ratio 1/1), MNP-TEOS-Am/Me 1/4 (MNP-TEOS surface grafted by amino- and methyl-functions at molar ratio 1/4), MNP-TEOS-Am/Me 1/16 (MNP-TEOS surface grafted by amino- and methyl-functions at molar ratio 1/16), MNP-TEOS-Me (MNP-TEOS surface grafted by pure methyl-functions).

2. Results of HPLC-DAD-MS Analysis for Biomimetic Oxidation of Amlodipine (1)

2.1. HPLC-DAD Chromatograms of the Biomimetic Reactions

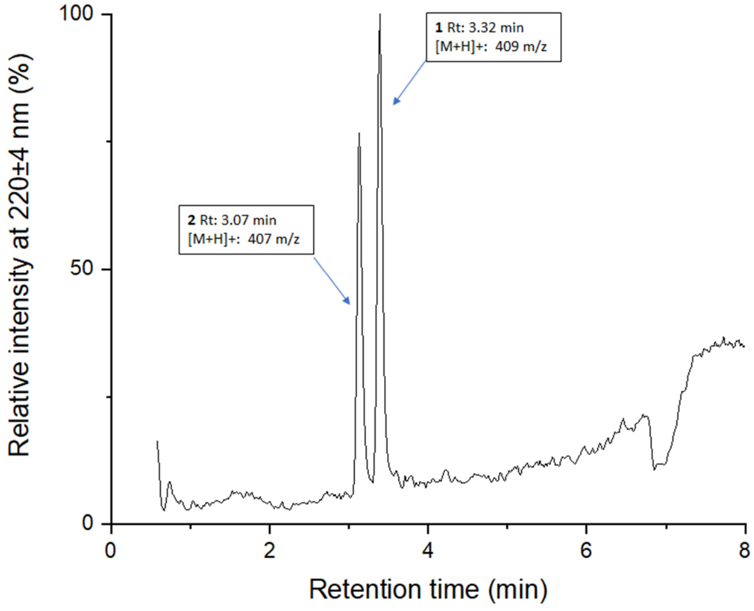

Figure S4. Representative HPLC data of FeTPFP-MNPs-catalyzed biomimetic oxidation of amlodipine (**1**) to major in vivo didehydro metabolite (**2**) in batch mode.

2.2. Identification of Amlodipine and its Metabolites by Mass Spectroscopy (MS)

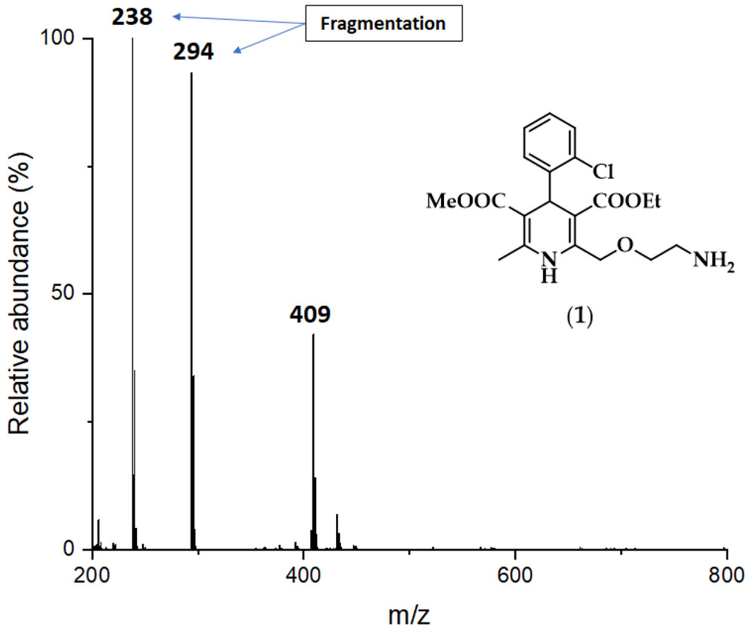

Figure S5. MS spectrum of amlodipine (**1**).

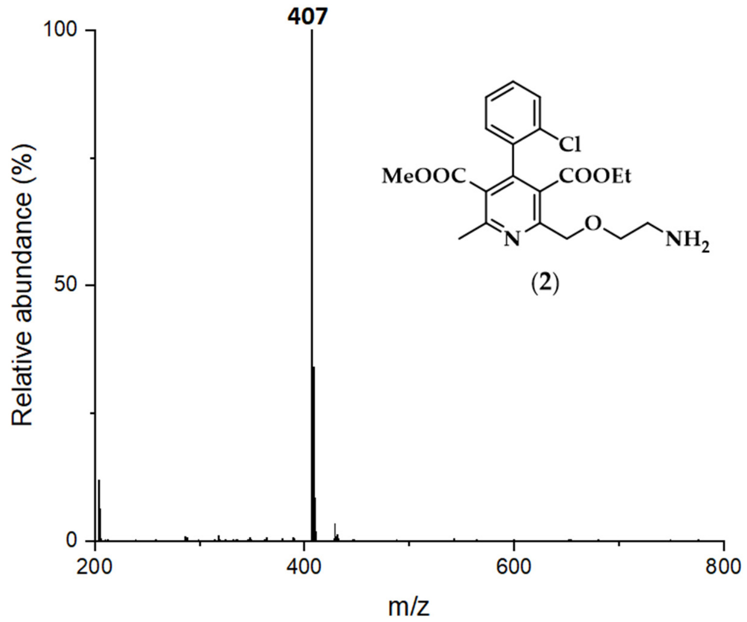

Figure S6. MS spectrum of amlodipine didehydro metabolite (**2**) as a main product of biomimetic oxidation.

3. Structural Identification of Amlodipine (1) and Amlodipine Metabolite (2) via HRMS Fragmentation and NMR.

3.1. Summary of Retention Time, Observed Parent- and Characteristic Fragment Ions of Amlodipine and Didehydro Amlodipine

Table S1. Summary of retention time, observed parent- and characteristic fragment ions of amlodipine and didehydro amlodipine.

Compound	RT (min)	$[M+H]^+$ (*m*/*z*)	Mass error (ppm)	Collision Energy (eV)	Fragment ions (*m*/*z*)
Amlodipine	3.59	409.15276	0.69	20	377.12673, 294.08986, 238.06332[1]
Dehydro-amlodipine	3.22	407.13747	0.84	25	390.11102, 364.09539, 364.08483, 318.05351, 286.02732[1]

[1] most abundant fragment ion.

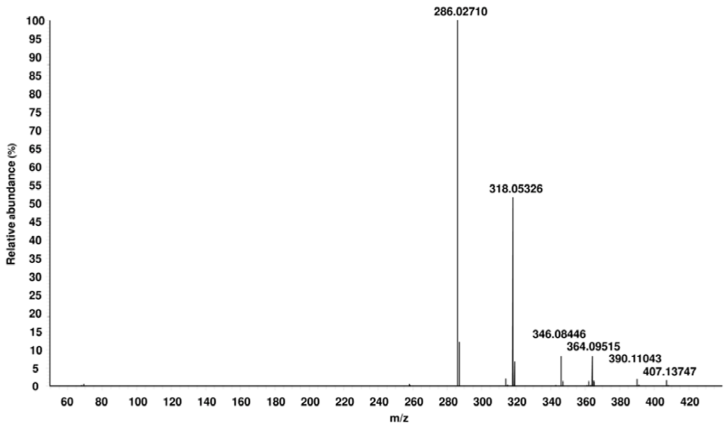

Figure S7. HRMS\MS spectrum of amlodipine didehydro metabolite (**2**) as a main product of biomimetic oxidation.

3.2. NMR Spectra of Didehydro Amlodipine Metabolite (2)

^{1}H NMR (500 MHz, CD_3-OD, δ ppm): 7.79-7.87 (2H, m, H-34, 33), 7.48 (1H, dd, J = 8 Hz, J = 1.3 Hz, H-21), 7.40 – 7.45 (4H, m, H-32, 31, 30, 19), 7.36 (1H, td, J = 7.5 Hz, J = 1.4 Hz, H-17), 7.18 (1H, dd, J = 7.6 Hz, J = 1.6 Hz, H-18), 4.80 – 4.84 (2H, m, H-9), 3.96 – 4.05 (2H, m, H-2), 3.70 (2H, t, J = 5.2 Hz, H-7), 3.53 (3H, s, H-10) 3.04 – 3.10 (2H, m, H-6) 2.63 (3H, s, H-15), 1.90 (56H, s, H39), 0.91 (2H, t, J = 7.2 Hz, H-1) ppm.

^{13}C NMR (126 MHz, CD_3-OD, δ ppm): 180. 3 (C-40) 168.5 (C-13), 167.9 (C-14), 157.8 (C-28), 157.0 (C-24), 46.8 (C-25), 146.4 (C-35), 136.1 (C-20), 134.1 (C-22), 131.8 (C-18), 131.7 (C-30), 131.5 (C-31, 32), 130.5 (C-21), 130.3 (C-27) 129.4 (C-19), 128.3 (), 128.1 (C-23), 127.7 (C-17), 127.1 (C-34, 33), 73.5 (C-9), 69.0 (C-7), 63.0 (C-2), 53.0 (C-10), 40.9 (C-6), 24.2 (C-39), 23.1 (C-15), 14.0 (C-1)
